# Factors predicting survival after post-transplant hepatocellular carcinoma recurrence

**DOI:** 10.1007/s00534-012-0528-4

**Published:** 2012-06-19

**Authors:** Christian Toso, Sonia Cader, Ariane Mentha-Dugerdil, Glenda Meeberg, Pietro Majno, Isabelle Morard, Emiliano Giostra, Thierry Berney, Philippe Morel, Gilles Mentha, Norman M. Kneteman

**Affiliations:** 1 Divisions of Transplant and Abdominal Surgery, Department of Surgery, University of Geneva Hospitals, Rue Gabrielle-Perret-Gentil, 1211 Geneva, Switzerland; 2 Division of Transplantation, Department of Surgery, University of Alberta, 2D4.44 Mackenzie Center, Edmonton, AB T6G 2B7 Canada; 3 Division of Gastro-enterology, Department of Internal Medicine, University of Geneva Hospitals, Rue Gabrielle-Perret-Gentil, 1211 Geneva, Switzerland

**Keywords:** Hepatocellular carcinoma, Recurrence, Survival

## Abstract

**Background:**

Although factors associated with an increased risk of recurrence after liver transplantation for hepatocellular carcinoma (HCC) have been extensively studied, the history of patients with a post-transplant recurrence is poorly known.

**Methods:**

Patients experiencing a post-transplant HCC recurrence from 1996 to 2011 in two transplant programs were included. Demographic, transplant, and post-recurrence variables were assessed.

**Results:**

Thirty patients experienced an HCC recurrence–22 men and 8 women with a mean age of 55 ± 6 years. Sixteen (53 %) were outside the Milan criteria at the time of transplantation. Most recurrences (60 %) appeared within the first 18 months after transplantation, ranging between 1.7 and 109 months (median 14.2 months). Mean post-recurrence survival was 33 ± 31 months. On univariate analysis, total tumor volume (TTV; *p* = 0.047), microvascular invasion (*p* = 0.011), and time from transplant to recurrence (*p* = 0.001) predicted post-recurrence survival. On multivariate analysis, both time from transplant to recurrence (*p* = 0.001) and history of rejection (*p* = 0.043), but not the location of the recurrence or the type of recurrence treatment, predicted post-recurrence survival.

**Conclusion:**

This study suggests that patients with early post-transplant HCC recurrence have worse outcomes. Those with a history of graft rejection have better survivals, possibly due to more active anti-cancer immunity.

## Introduction

Liver transplantation is the best treatment option for patients with early hepatocellular carcinoma (HCC). It offers better outcomes than competing strategies, with expected five-year survivals of 70–90 % [1–3]. Despite these good results, some 10 % of patients experience a post-transplant HCC recurrence, which leads to death in most cases.

The risk of post-transplant recurrence has been extensively studied, with the proposal of various candidate selection criteria including a wide range of extension compared with the classical Milan criteria [1–3]. Overall, it appears that both the tumor burden (assessed as the total tumor volume [TTV] or the maximum tumor size) and biological factors, such as alpha fetoprotein (AFP), best predict outcome, and these factors can help maintain low rates of post-transplant recurrence.

While recurrence is a relatively rare event, only a few publications with limited sample sizes have looked at this specific group of patients. Careful study of this group holds the potential for improvement in their management. The present study assesses the history and risk factors predicting survival after post-transplant HCC recurrence in a large cohort combining patients at two centers with similar transplant candidate selection criteria and outcomes.

## Patients and methods

### Patient inclusion

This study retrospectively assessed liver transplant recipients with a post-transplant HCC recurrence established by typical imaging and/or biopsy. Only patients transplanted after the publication of the Milan criteria were included (December 1996–April 2010) [1]. None of them had an incidental HCC on the explanted liver. Patients were transplanted at the University of Geneva, Switzerland, or at the University of Alberta, Edmonton, Canada. The study was approved by the institutional ethics review board committees of both institutions.

### Transplant characteristics and recurrence assessment

Both programs used extended inclusion criteria. At the University of Geneva, patients within Milan or downstaged to Milan were all considered for transplantation. At the University of Alberta, selection criteria were a single HCC ≤7.5 cm in diameter, or multiple tumors (without number restriction) ≤5 cm. Patients with a biopsy-proven poorly differentiated HCC larger than 5 cm were excluded in the initial Alberta series. Beginning in 2010 (Geneva) and 2007 (Alberta), a score based on a combination of TTV (≤115 cm^3^) and AFP (≤400 ng/ml) was used for candidate selection. With the use of such a score, similar outcomes can be achieved within Milan, and beyond Milan, but within the TTV/AFP score [2, 4, 5]. The TTV was obtained by adding the maximum volume of each HCC computed based on the formula (4/3)π*r* ^3^, calculated using the maximum radius of each tumor. Of note, the TTV does not include any strict limit in the number of HCCs, and two patients with 20 lesions have been included (both alive and well 3 and 6 years after transplantation). In the present study, pathological HCC characteristics were used for TTV calculation. All candidates with large liver vessel HCC invasion or with extrahepatic disease were excluded. Beginning in 2002 (Geneva) and 1996 (Alberta), immunosuppression was sirolimus-based starting from the time of transplantation [6, 7].

Post-transplant monitoring of recurrence was performed by thoraco-abdominal computed tomography (CT) scan every 6 months for the first 3 years and by ultrasonography (US) every 6 months thereafter. After the third post-transplant year, extrahepatic recurrences were detected on the basis of symptoms.

### Analyses and statistical methods

The demographics of patients with recurrence were assessed and factors predicting post-recurrence survival determined. Rejection was designated to include biopsy-proven rejections and events requiring treatment for rejection (even if not biopsy-proven). Prospectively established databases were used retrospectively (OTTR; Hickman-Kenyon Systems, Omaha, NE, USA). Survival was analyzed by the Kaplan–Meier method and differences between groups tested by log-rank or Cox tests. Multivariate analysis using a Cox proportional hazards model was used for the assessment of the prognostic factors reaching *p* values of at least 0.2 on univariate analysis. Further tests included the χ^2^ for categorical variables and *t*-test for continuous variables. A standard alpha level of 0.05 indicated statistical significance. Results are expressed as means ± standard deviation (SD). Analyses were conducted using SPSS 15.0 (SPSS, Chicago, IL, USA).

## Results

### Demographics

During the study period, 234 liver transplantations were performed for HCC and 30 recipients experienced a recurrence (overall rate of recurrence: 12.8 %; 12/97, 12.4 % in Geneva and 18/137, 13.1 % in Alberta). The recipients with recurrence were eight women and 22 men, with a mean age of 55 ± 6 years (Table 1). The most frequent underlying liver disease was linked to hepatitis C virus (HCV) infection. Pre-transplant local HCC treatment included transarterial chemoembolization (*n* = 14), surgery (*n* = 6), percutaneous alcohol injection (*n* = 4), radio-frequency ablation (*n* = 3), and cryo-ablation (*n* = 1). All patients with recurrence had received whole liver grafts, except for one live liver graft recipient. Mean follow up after recurrence was 32.9 ± 31.2 months [median 18.7 (1.7–110.2) months].

**Table 1:** Patient and tumor characteristics at transplantation

Patients (number)	30
Mean age (years ± SD)	55 ± 6
Gender	Female 8/male 22
Cause of liver disease (%)	
HCV (±alcohol, ±HBV)	23 (77)
HBV	5 (17)
Alcohol	1 (3)
Alpha 1 anti-trypsin	1 (3)
MELD score	12 ± 6.5
Number of HCCs	4.3 ± 7.8
Largest HCC (cm ± SD)	3.8 ± 1.8
Total tumor volume (cm^3^ ± SD)	58 ± 69
Patients with total tumor volume ≥115 cm^3^	5 (17)
Alpha fetoprotein (AFP; ng/ml ± SD)	295 ± 711
Patients with AFP ≥400 ng/ml	4 (13)
Macrovascular invasion (%)	9 (30)
HCC grade 1/2/3 (%)	2 (8)/13 (50)/11 (42)
Within Milan criteria (%)	15 (50)
mTOR inhibitor-based immunosuppression	19 (63)

*HCC* hepatocellular carcinoma, *HCV* hepatitis C virus infection, *HBV* hepatitis B virus infection, *MELD* model for end-stage liver disease, *mTOR* mammalian target of rapamycin

In an effort to understand the cause of recurrence, the profile of each patient with recurrence was assessed independently. Half of the patients were outside the Milan criteria, five (17 %) presented a TTV >115 cm^3^ and four (13 %) an AFP >400 ng/ml. In addition, nine patients (30 %) showed a macrovascular invasion on pathology, 18 (60 %) a microvascular invasion and 11 (37 %) a poorly differentiated HCC. These tumor factors could explain all recurrences except four. In these four patients, smoking was the only factor potentially contributing to recurrence. While none of the patients with recurrence had been actively smoking at the time of transplantation, these four patients all had a past history of heavy smoking, at 15, 20, 30, and 30 pack-years. Of all the studied patients, 16 patients (53 %) had a history of smoking (mean 26.5 ± 10 pack-years, range 10–50 pack-years). Despite aggressive pre-transplant anti-smoking policies and support programs at both centers, nine recipients were still actively smoking at the time of transplantation.

### Recurrence characteristics

**Fig. 1:**
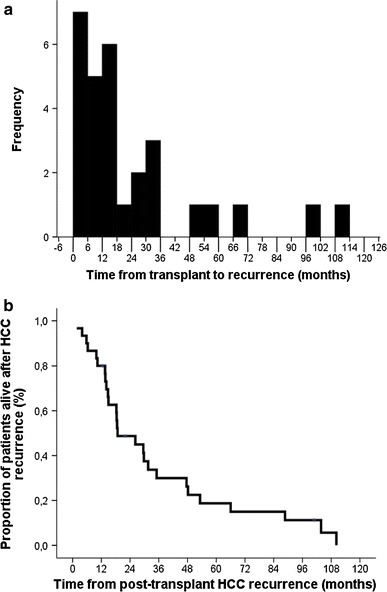
**a** Histogram showing the distribution of the time between transplantation and recurrence in the 30 studied patients. **b** Overall post-recurrence survival (median survival 18.8 ± 6.8 months). *HCC* Hepatocellular carcinoma

Recurrence appeared as early as 1.7 and up to 109 months after transplantation (mean 24 ± 28 months). Most of them occurred within 18 months (median 14.2 months, Fig. 1a), while recurrence was an unusual event after 36 months (5/30 or 16.7 %). Recurrence most often involved the liver, lungs, and bones, and was multiple in eight patients (27 %, Table 2). Of note, the patient with the earliest recurrence, 1.7 months after transplantation, presented with multiple poorly differentiated HCC metastases in the liver graft.

**Table 2:** HCC recurrence characteristics

Time from transplant to recurrence (months ± SD)	24 ± 28
Patients alive with recurrence (%)	3 (10)
Time from recurrence to death (months ± SD)	33 ± 31
Location of recurrence	
Liver	14
Lung	13
Bone	7
Other	4
Multiple locations (%)	8 (27)
Treatment of recurrence	
Medical/palliation	21 (70)
Loco-regional (including TACE, PEI)	3 (10)
Surgical resection	6 (20)

*TACE* transarterial chemo-embolisation, *PEI* percutaneous ethanol injection

Recurrence was treated by surgery in 20 % of patients (Table 2), but most underwent loco-regional (10 %) or palliative (70 %) treatments. Mean post-recurrence survival was 33 ± 31 months (median survival 18.8 ± 6.8 months), with deaths happening between 1.7 and 110.2 months after recurrence (Fig. 1b).

### Factors predicting survival after post-transplant HCC recurrence

**Fig. 2:**
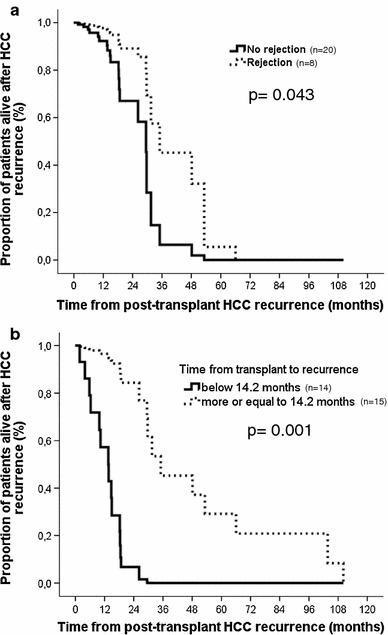
Post-recurrence survival according to the occurrence of a rejection during the first 6 months after transplantation (**a**) and according to the time between transplantation and the recurrence (**b**)

On univariate assessment, post-recurrence survival was predicted by tumor factors (TTV and microvascular invasion) and the time between transplantation and recurrence (Table 3). On multivariate analysis, the time between transplant and recurrence [hazard ratio; HR 0.88 (95% confidence interval; CI 0.81–0.95), *p* = 0.001] and the occurrence of a rejection during the first 6 months after transplantation [HR 3.46 (95% CI 1.04–11.56), *p* = 0.043] predicted post-recurrence survival (Fig. 2). In order to better estimate the impact of these two variables, independent survival curves were drawn (using the median time between transplant and recurrence of 14.2 months as cut-off (Table 2). A late diagnosis of recurrence and the occurrence of a rejection during the first 6 months after transplantation were both associated with longer survivals. Of note, the extent of the recurrence (single vs. multiple sites) and the type of recurrence treatment did not alter survival. Two patients were put on sorafenib after recurrence.

**Table 3:** Factors predicting survival after post-transplant HCC recurrence

Variables^a^	Univariate analysis		Multivariate analysis^a^	
	HR (95 % CI)	*p*	HR (95 % CI)	*p*
Age at transplant	1.02 (0.96–1.09)	0.55		
Cause of liver disease				
HCV (±alcohol, ±HBV)	1			
HBV	2.66 (0.33–21.21)	0.36		
Smoking (pack-years)	1.01 (0.97–1.04)	0.74		
Transplant characteristics				
Year of transplantation	0.55 (0.20–1.47)	0.22		
Pre-transplant local HCC treatment (yes vs. no)	0.55 (0.20–1.47)	0.23		
MELD at transplant	1.01 (0.96–1.05)	0.88		
Number of HCCs	1.05 (0.98–1.11)	0.22		
Total tumor volume	1.01 (1.000–1.012)	0.047		
Alpha fetoprotein	1 (0.999–1.001)	0.93		
Microvascular invasion (yes vs. no)	3.07 (1.29–7.28)	0.011		
HCC grade (1 or 2 vs. 3)	0.76 (0.32–1.79)	0.53		
Within Milan criteria (within vs. beyond)	0.56 (0.26–1.23)	0.15		
mTOR inhibitor-based immunosuppression (yes vs. no)	0.98 (0.45–2.15)	0.96		
Rejection from 0 to 6 months post-transplant (yes vs. no)	1.9 (0.75–4.83)	0.18	3.46 (1.04–11.56)	0.043
Recurrence characteristics and treatment				
Time from transplant to recurrence	0.91 (0.86–0.96)	0.001	0.88 (0.81–0.95)	0.001
Location of recurrence				
Liver	1			
Lung	1.01 (0.22–4.69)	0.99		
Other	1.12 (0.23–5.44)	0.89		
Surgical resection (yes vs. no)	1.31 (0.44–3.9)	0.62		

*HR* hazard ratio, *CI* confidence interval, *HCV* hepatitis C virus infection, *HBV* hepatitis B virus infection

^a^Significant variables

## Discussion

The present study suggests that the time of appearance of a post-transplant HCC recurrence has a strong impact on survival and that the immune status (history of rejection) may also play a role.

A post-transplant recurrence can appear in two situations; firstly, when an extra-hepatic metastasis has been missed (or was not detectable) during the pre-transplant work-up. Secondly, a recurrence can also be the consequence of circulating HCC cells engrafting and growing in a target organ during the peri-transplant period [8]. Given the higher original cancer load of the first mechanism, such recurrences are expected to appear earlier after transplantation. These two mechanisms may help explain the observed bimodal distribution of recurrences, with most of them appearing during the first 18 months and some, more indolent, diagnosed up to 10 years after transplantation (Fig. 1a).

Along the same line, patients diagnosed with early recurrence may not only have a higher original cancer load, but may also have a more aggressive biology. Perhaps as a result of these factors, these patients have a significantly lower life expectancy. This observation reinforces previous studies suggesting that the time between transplant and recurrence is key in predicting the outcome after recurrence, with worse survival rates when recurrence is diagnosed within 12 months from transplantation [9–11]. Such data are important to guide the management of and to better inform patients.

The observed overall post-recurrence survival was close to 3 years on average (median survival 18.8 ± 6.8 months), which is similar to previously reported data after whole organ liver transplantation (survivals ranging from 8 to 24 months in average [9–13]).

In the present study, the occurrence of a rejection during the first 6 months after transplantation was also an independent predictor of post-recurrence survival. This finding may reflect the use of a less profound immunosuppression combination, more active immunity, and potentially the presence of active cytotoxic immune cells in patients demonstrating a rejection. These factors illustrate the yin-yang situation of liver transplantation for hepatocellular carcinoma (HCC), which is performed in the presence of both donor (allogeneic) and cancer antigens. While the allogeneic immunity should be decreased, the anti-cancer one should be preserved and potentially enhanced. Along this line, stronger immune depletion (as assessed by low ATP production by peripheral blood mononuclear cells, determined using the ImmuKnow test, Cylex, Columbia, MD) has been associated with a higher risk of post-transplant HCC recurrence [14]. Similarly, the risk of HCC recurrence has been strongly correlated with the level of calcineurin inhibitor immunosuppression and with the use of antilymphocyte antibody induction [15, 16]. While further validation is mandatory, such data would promote the use of milder immunosuppression combinations in patients undergoing transplantation for HCC.

Surgery has been reported to significantly improve the post-recurrence survival of patients with local recurrence [9, 10, 12, 17, 18]. While this was not the case in the present study (potentially due to the relatively low sample size), a surgical resection should be attempted whenever feasible, as supported by the report of a recent consensus conference [19]. Of note, liver re-transplantation is currently recognized as not appropriate after post-transplant HCC recurrence [19].

Although randomized data are still pending, several studies suggest an anti-cancer effect and better post-transplant survivals with the use of mammalian target of rapamycin (mTOR) inhibitors, and patients may be put on such drugs when a recurrence occurs [6, 7, 19, 20]. Of note, the observed lack of improved post-recurrence survival with mTOR inhibitors in the present study may be related to the high patient heterogeneity and to the relatively low sample size.

Of note, most of the patients in the present study presented risk factors for post-transplantation recurrence, but four had small HCCs and low AFP levels. In these four patients, smoking was the only contributory factor. While this observation clearly requires further exploration, it reinforces the need to promote and support cessation of smoking prior to liver transplantation for HCC. As a reminder, smoking has been associated with the occurrence of de-novo HCC, with close to half of diagnosed patients demonstrating a past or current smoking history [21]. Active smoking has been reported to be more strongly associated with HCC than obesity or heavy alcohol intake (odds ratio of 4.55 vs. 2.13 and 1.77) [21]. Similarly, smoking has been associated with an increased risk of colon cancer metastasis and recurrence after surgery, even in past smokers [22, 23].

The present study suggests that early recurrence and the use of more profound immunosuppression result in worse post-recurrence outcomes. Such data can be used to better manage and inform recipients of liver transplantation for HCC prior to and after a potential recurrence.
